# Anxiety and Comorbidities Differences in Adults with Chronic Pulmonary Diseases: Serbian Single Center Experience

**DOI:** 10.3390/medicina58030392

**Published:** 2022-03-06

**Authors:** Natasa Mujovic, Kristina Popovic, Jelena Jankovic, Snezana Popovac Mijatov, Nebojsa Mujovic, Jelena Bogdanovic, Mihailo Stjepanovic, Ljubica Nikcevic, Natasa Radosavljevic, Dejan Nikolic

**Affiliations:** 1 Faculty of Medicine, University of Belgrade, 11000 Belgrade, Serbia; natashamujovic68@gmail.com (N.M.); jjelena1984@gmail.com (J.J.); nmujovic@gmail.com (N.M.); jeca.bogdanovic@yahoo.com (J.B.); mihailostjepanovic@gmail.com (M.S.); 2 Center for Physical Medicine and Rehabilitation, University Clinical Center of Serbia, 11000 Belgrade, Serbia; popovickristina@ymail.com (K.P.); popovacsnezana@gmail.com (S.P.M.); 3 Clinic for Pulmonary Diseases, University Clinical Center of Serbia, 11000 Belgrade, Serbia; 4 Clinic for Cardiology, University Clinical Center of Serbia, 11000 Belgrade, Serbia; 5 Clinic for Endocrinology, University Clinical Center of Serbia, 11000 Belgrade, Serbia; 6 Hospital for Cerebrovascular Diseases “Sveti Sava”, 11000 Belgrade, Serbia; ljubicanikcevic@yahoo.com; 7 Department of Physical Medicine and Rehabilitation, King Abdulaziz Specialist Hospital, Taif 26521, Saudi Arabia; dr.natasa.radosavljevic@gmail.com; 8 Department of Physical Medicine and Rehabilitation, University Children’s Hospital, 11000 Belgrade, Serbia

**Keywords:** chronic pulmonary disease, lung cancer, anxiety, comorbidities

## Abstract

*Background and objectives*: The purpose of this study is to investigate the differences in the degree of the anxiety and comorbidity levels in patients with different chronic pulmonary diseases such as chronic obstructive bronchitis (COPD) without emphysema phenotype, pulmonary emphysema, bronchial asthma and lung cancer. *Materials and Methods*: The prospective clinical study included 272 patients that were diagnosed and treated of pulmonary pathology. COPD (without emphysema phenotype) (Group-1), pulmonary emphysema (Group-2), bronchial asthma (Group-3) and lung cancer (Group-4) were assessed. For the evaluation of the anxiety degree, we used Hamilton Anxiety Rating Scale (HAM-A). *Results*: The degree of cardiovascular symptoms was significantly higher in Group-1 versus Group-2 (*p* < 0.001), Group-3 (*p* = 0.001) and Group-4 (*p* = 0.013), and significantly higher in Group-4 versus Group-2 (*p* = 0.046). The degree of respiratory symptoms was significantly higher in Group-1 versus Group-2 (*p* < 0.001), Group-3 (*p* < 0.001) and Group-4 (*p* = 0.002), and significantly higher in Group-4 versus Group-2 (*p* = 0.013) and versus Group-3 (*p =* 0.023). For gastrointestinal symptoms, the degree of one was significantly higher in Group-1 versus Group-2 (*p* < 0.001), Group-3 (*p* < 0.001) and Group-4 (*p* = 0.017). Somatic subscale values were significantly higher in Group-1 versus Group-2 (*p* < 0.001), Group-3 (*p* < 0.001) and Group-4 (*p* = 0.015), and significantly higher in Group-4 versus Group-2 (*p* = 0.024). Total HAM-A score was significantly higher in Group-1 versus Group-2 (*p* = 0.002) and Group-3 (*p* = 0.007). *Conclusions*: Patients with COPD (without emphysema phenotype) followed by the lung cancer are at elevated risk of being more mentally challenged in terms of increased anxiety. Furthermore, patients with exacerbation of evaluated pulmonary pathologies have various levels of comorbidities degrees.

## 1. Introduction

Chronic diseases are long-term progressive conditions that develop slowly and last for a long period time. In general, patients with any chronic disease have a higher susceptibility of being psychologically and emotionally challenged [1]. Sastre et al. pointed out that patients with respiratory diseases compared with the general population have greater levels of psychiatric comorbidity [2]. Previously, numerous studies have examined the anxiety in patients with a chronic disease [2,3,4,5]. It was noted that about every third patient with chronic obstructive pulmonary disease (COPD) had anxiety symptoms [4]. Furthermore, these patients with present anxiety are more susceptible to exacerbations as well as hospitalizations [5]. In the systematic review and meta-analysis of Ye et al., [6], it was noted that the anxiety can be considered to be the strongest predictor of breathlessness along with its negative impact on cognition and coping behavior in asthmatic patients. The connection between anxiety and asthma is complex and multidimensional with possible mechanisms that might include the role of hypoxia and hypercapnia on neural circuits controlling fear responses sensitization, pro-inflammatory cytokines where certain interleukins (IL) such as IL-6 and IL-10 could be associated with emotional disturbances including anxiety [6].

The systematic review and meta-analysis of Wang et al. [7] stated that 10% of cancer patients have anxiety disorder. In the study of Polanski [8], it was noted that 16–23% of patients with lung cancer are diagnosed with fear and anxiety. There are several proposed possible mechanisms among them endocrinological and immunological that could lead to the better understanding of anxiety impact on cancer outcomes. Additionally, maladaptive lifestyle habits in individuals with anxiety could be indirectly associated with the cancer [7]. Furthermore, it was shown that cancer type and cancer stage might be the factors that could influence the prevalence of anxiety [9].

In previous reports, numerous comorbidities were associated with COPD [10,11,12]. In the study of Negewo et al., psychiatric, respiratory, cardiovascular and gastrointestinal diseases were evaluated as well as metabolic syndrome and osteoporosis [10]. Moreover, it was proposed that the COPD is associated with a low-grade systemic inflammation with a production of pro inflammatory markers that could alter development and progression of the cardiovascular diseases in COPD patients [11]. Regarding the gastrointestinal disease it was pointed that the chronic systemic infections, for instance with Helicobacter pylori, might be associated with the development and progression of COPD [11].

For the patients with asthma, it was shown that numerous comorbidities (respiratory and psychological conditions, cardiovascular and gastrointestinal diseases, obesity, hormonal and metabolic disorders, infections) could be associated with them [13,14]. It should be stated as well that certain comorbidities such as obesity could affect the response to the asthma medications and might increase other comorbidities such as obstructive sleep apnea, depression and other [15].

For the lung cancer patients, cardiovascular comorbidities are among the most frequent comorbidities [16]. Further comorbidities that were described include diabetes, cerebrovascular and respiratory diseases [16]. In the study of Xiu et al., it was noticed that certain comorbidities such as hypertension and diabetes mellitus type 2 could be associated with worse prognosis in patients with extensive-stage small-cell lung carcinoma [17].

The purpose of this study is to investigate the differences in the degree of the anxiety and comorbidity levels in patients with different chronic pulmonary diseases such as chronic obstructive pulmonary disease (COPD) without emphysema phenotype, pulmonary emphysema, bronchial asthma and lung cancer.

## 2. Materials and Methods

### 2.1. Study Group

The prospective clinical study that was conducted in a single center included 272 patients that were diagnosed and treated of pulmonary pathology. The eligible participants were those with one of four referral diagnoses: COPD (without emphysema phenotype), pulmonary emphysema, bronchial asthma and lung cancer. Diagnoses were made by board certified specialist of Internal medicine with experience in pulmonary pathology. Every patient was referred to adequate diagnostic procedure in order to confirm diagnosis. The exclusion criteria were other pulmonary diseases. The study sample was divided into four groups: Group-1 with COPD (without emphysema phenotype); Group-2 with pulmonary emphysema; Group-3 with bronchial asthma; Group-4 with lung cancer.

Frequencies of further patient characteristics were assessed: smoking, hypertension, obesity. Female and male gender as well as age of participants were also presented.

### 2.2. Anxiety Assessment

For the evaluation of the degree of anxiety, we used the Hamilton Anxiety Rating Scale (HAM-A). It has good psychometric properties, and is one among the most widely used scales in research anxiety severity evaluation [18]. HAMA-A has 14 questions and measures psychological and somatic symptoms [18,19]. Every question was graded from 0 to 4, where 0 is for no symptoms; 1 for mild symptoms; 2 is for moderate; 3 is for severe; and 4 is for very severe symptoms [19]. In our study we assessed separately: psychic component with psychic subscale (questions 1–6 and question 14) and somatic component by somatic subscale (questions 7–13) [20].

The interview was performed by an experienced board-certified Physical Medicine and Rehabilitation specialist and University Professor with more than 20 years of experience in rehabilitation filed of pulmonary medicine at the University Clinical Center.

### 2.3. Statistical Analysis

The results were presented as mean values (MV) with standard deviation (SD). For the tested questionnaire, 95 confidence intervals (CI) were calculated. To test the normal distribution, we performed a Kolmogorov–Smirnov test of normality. A Mann–Whitney U test was conducted for samples without normal distribution to estimate the statistical significance between two groups with different diagnoses. For estimation of statistical significance among four groups with different diagnoses without normal distribution, we used a Kruskal–Wallis test, whereas for those with normal distribution we used a one-way ANOVA. A post hoc Tukey test was used to determine statistical significance between tested groups after One way ANOVA test. To evaluate the correlation between tested questions and total score of the questionnaire with certain diagnoses we used Point-Biserial Correlation (*r* value), where dichotomous variable was diagnosis and continuous variable were test questions and total score of the questionnaire. The statistical significance was set at *p* < 0.05.

## 3. Results

Frequencies of specific characteristics of the patients that were included in the study were presented in Table 1. Smoking was most frequent in Group 1 (91.7%), Group 2 (90.5%) and Group 4 (90%). Hypertension was most frequent in Group 3 (63.2%).

The presence and degree of intellectual dysfunction (*p =* 0.031), muscular somatic complaints (*p =* 0.031), cardiovascular symptoms (*p* < 0.001), respiratory symptoms (*p* < 0.001) and gastrointestinal symptoms (*p* < 0.001) significantly differed among tested groups. Additionally, both psychotic subscale values (*p =* 0.029) and somatic subscale values (*p* < 0.001) as well as total HAM-A score (*p* < 0.001) significantly differed among tested groups (Table 2).

Comparisons between HAM-A questions, subscales and total score in different pathologies were presented in Table 3. When compared between two groups of patients with different diagnoses, presence and degree of intellectual dysfunction was significantly higher in Group-1 versus Group-2 (*p =* 0.003) and versus Group-3 (*p =* 0.047). Regarding presence and degree of somatic muscular complaints, it was significantly higher in Group-1 versus Group-2 (*p =* 0.002), as well in Group-4 versus Group-2 (*p =* 0.038), for cardiovascular symptoms it was significantly higher in Group-1 versus Group-2 (*p* < 0.001), versus Group-3 (*p =* 0.001) and versus Group-4 (*p =* 0.013), and significantly higher in Group-4 versus Group-2 (*p =* 0.046). The presence and degree of respiratory symptoms was significantly higher in Group-1 versus Group-2 (*p* < 0.001), versus Group-3 (*p* < 0.001) and versus Group-4 (*p =* 0.002), and significantly higher in Group-4 versus Group-2 (*p =* 0.013) and versus Group-3 (*p =* 0.023). For gastrointestinal symptoms, the presence and degree of one was significantly higher in Group-1 versus Group-2 (*p* < 0.001), versus Group-3 (*p* < 0.001) and versus Group-4 (*p =* 0.017). Somatic subscale values were significantly higher in Group-1 versus Group-2 (*p* < 0.001), versus Group-3 (*p* < 0.001) and versus Group-4 (*p =* 0.015), and significantly higher in Group-4 versus Group-2 (*p =* 0.024). Regarding total HAM-A score, it was significantly higher in Group-1 versus Group-2 (*p =* 0.002) and versus Group-3 (*p =* 0.007).

The COPD (without emphysema phenotype) significantly positively correlated with insomnia (*r =* 0.15; *p =* 0.011), intellectual dysfunction (*r =* 0.21; *p* < 0.001), depressed mood (*r =* 0.17; *p =* 0.005), somatic muscular complaints (*r =* 0.19; *p =* 0.001), cardiovascular symptoms (*r =* 0.32; *p* < 0.001), respiratory symptoms (*r =* 0.41; *p* < 0.001), gastrointestinal symptoms (*r =* 0.31; *p* < 0.001), genitourinary symptoms (*r =* 0.15; *p =* 0.014), autonomic symptoms (*r =* 0.14; *p =* 0.018), psychic subscale (*r =* 0.18; *p =* 0.003), somatic subscale (*r =* 0.34; *p* < 0.001) and total HAM-A score (*r =* 0.29; *p* < 0.001) (Table 4).

The pulmonary emphysema significantly negatively correlated with insomnia (*r =* −0.12; *p =* 0.047), intellectual dysfunction (*r =* −0.15; *p =* 0.011), somatic muscular complaints (*r =* −0.20; *p* < 0.001), somatic sensory complaints (*r =* −0.13; *p =* 0.028), cardiovascular symptoms (*r =* −0.23; *p* < 0.001), respiratory symptoms (*r =* −0.29; *p* < 0.001), gastrointestinal symptoms (*r =* −0.21; *p* < 0.001), autonomic symptoms (*r =* −0.06; *p =* 0.007), somatic subscale (*r =* −0.27; *p* < 0.001) and total HAM-A score (*r =* −0.21; *p* < 0.001) (Table 4).

The bronchial asthma significantly negatively correlated with cardiovascular symptoms (*r =* −0.12; *p =* 0.046), respiratory symptoms (*r =* −0.19; *p =* 0.002), gastrointestinal symptoms (*r =* −0.12; *p =* 0.045). None of tested HAM-A questions, subscales and total score significantly correlated with malignant pulmonary neoplasm (Table 4).

## 4. Discussion

This study has a purpose of rising the awareness of psychic disturbances that are associated with pulmonary diseases. Our results have brought the importance of psychic problems, anxiety as well as somatic disturbances that are present in these patients to our existing knowledge.

We have demonstrated that for a predominant proportion of patients with tested pulmonary diseases, except for those with bronchial asthma, smoking was present as an activity. Since smoking affects further deterioration of pulmonary function in patients with COPD or emphysema [21,22], they should be informed about the importance of the smoking cessation in order to reduce the disease progression.

In our study, it was shown that COPD (without emphysema phenotype) patients versus other evaluated groups with exacerbation had most severe anxiety levels, whereas those with pulmonary emphysema exacerbation were with lowest degree of anxiety. Both psychic and somatic levels were highest for those with COPD (without emphysema phenotype) exacerbation, whereas psychic levels were lowest for patients with asthmatic exacerbations and somatic levels lowest for those with pulmonary emphysema exacerbations. Furthermore, our study stressed that no significant differences were present between tested groups in psychic levels, whereas somatic levels were significantly increased in COPD (without emphysema phenotype) group versus other tested groups and for those with the lung cancer compared with pulmonary emphysema. Such differences could be explained to the certain degree by individual (intrinsic and/or environmental) factors associated with the evaluated respiratory disease or a condition.

The importance of the anxiety particularly in conditions where the disease has its progression course should be additionally evaluated. This is due to the fact that not so rarely in professional practice, patients are just informed about the presence of cancer or the progression of the one without dedicating the time to inform them about its impact on everyday activities. It should be stressed that chemotherapy with side effects or the disease progression with amplified symptomatology including but not limited to the pain or various degrees of asphyxia could additionally challenge their anxiety levels. From our experience fear of unknown, fear of failure and even the death might present the important negative factors in further treatment of these patients.

Proper and timely recognition of the present comorbidities are important since they can be the causable factors of certain conditions that might be misinterpreted as the exacerbation of the primary pulmonary disease. Particularly the dyspnea as the consequence or symptom of the heart failure, anemia or obesity could be misinterpreted as the progression of the COPD [22,23]. In our study just above every third patient was obese in groups with COPD (without emphysema phenotype) and bronchial asthma.

Unlike asthma, which most often occurs in youth, COPD is more often diagnosed after the age of 40 [24]. For this reason, most patients have a many comorbidities including but not limited only to cardiovascular disease, obesity, and gastroesophageal reflux disease.

In previous studies, it was shown that the link between COPD and cardiovascular disease has potentially common risk factors, such as: smoking status, arterial hypertension, high cholesterol levels and obesity. Furthermore, inflammation with active cytokines has a potential to increase an oxidative stress and endothelial dysfunction in coronary heart disease [25]. The same mediators are involved in inflammatory reactions observed in COPD. Moreover, chronic pulmonary heart disease and pulmonary hypertension often develop in advanced COPD. In addition to endothelial dysfunction, pulmonary specific mechanisms also play a role in the development of pulmonary hypertension [26]. These mechanisms are vasoconstriction due to hypoxemia in advanced COPD or emphysema due to the destruction of the alveolar-capillary membrane, smoking which causes inflammation of the vascular wall. All this leads to the worsening of gas exchange, dyspnea, load on the right heart and heart failure [26]. Such observations might be to the certain degree in line with our study findings, particularly increased values of cardiovascular and respiratory symptoms in patients with COPD (without emphysema phenotype).

Corticosteroids are used as a symptomatic therapy in the worsening of COPD and the advanced stage of the lung cancer. They can cause gastrointestinal problems, with symptoms that might include abdominal pain, acid reflux, nausea or vomiting, abdominal distension, diarrhea, tenesmus and belching [27]. The hyperinflation that occurs in COPD affects the position of the organs in the abdomen and this can further affect the onset of gastrointestinal symptoms [28]. Smoking in COPD and lung cancer is higher than in asthma, who are mostly non-smokers, and this may be the cause of more frequent problems than in asthmatics. According to data from the literature, it is noted that there is significantly higher risk of ulcerative colitis and Crohn’s disease among COPD patients. This could be explained by the fact that those diseases may share common inflammatory pathways or compromised intestinal perfusion as a consequence of hypoxemia among COPD patients leading to ischemia [28]. High prevalence of gastro-oesophageal reflux disease in patients with COPD, which was associated with an increased risk of exacerbation and some drugs used to treat COPD as β2 agonists and theophylline preparations cause relaxation of the esophageal sphincter and potentiate the discomfort. These findings are in line with our results where we noticed that gastrointestinal symptoms were more severe in COPD (without emphysema phenotype) patients and in those with pulmonary carcinoma.

It is possible that the cause of more frequent problems with COPD, i.e., in patients with chronic bronchitis in relation to emphysema, is due to the fact that they have a chronic cough with expectoration, which is how they differ from emphysematics. Dyspnoea occurs in the advanced course of the disease with a higher degree of bronchial obstruction, and often the initial emphysema is proven only by a chest hair scanner when lung function is still largely preserved. Respiratory symptoms are also more common in patients with lung cancer due to the compression of the tumor in the airway, pain due to tumor infiltration or the appearance of superior vena cava syndrome.

Patients with lung cancer are often associated with anxiety and emotional problems, so that can lead to somatic symptoms [29]. In our study, we have demonstrated that somatic symptoms are second in line regarding severity degree, after COPD. Unforeseen and chronic events in COPD can lead to the anxiety that provokes somatic and emotional symptoms of anxiety or panic. Such patients are more likely to seek medical help, more likely to go for routine and unscheduled check-ups, more likely to be prescribed anti-inflammatory therapy, and have more hospital treatments as well as longer duration [30]. The higher the level of anxiety, the worse the control of the disease, and the worse the quality of life. According to the literature, the prevalence of panic attack is nearly ten times higher in people with COPD than in the general population [30]. Mental health problems with COPD are associated with a reduced ability to cope with the disease itself, which is progressive, declining lung function and more pronounced dyspnea, poorer quality of life, more frequent exacerbations and hospitalizations, poor adherence to treatment [30].

There are several limitations to this study. First one refers to the single center study. Second one refers to the study sample on one population (Serbian). Furthermore, a small number of study participants might be additional limiting factor in critical interpretation of obtained findings, thus further studies are needed on larger sample of participants.

## 5. Conclusions

Our results have demonstrated that patients with COPD (without emphysema phenotype) followed by lung cancer are at an elevated risk of being more mentally challenged in terms of increased anxiety. Furthermore, we have shown that patients with exacerbation of evaluated different pulmonary pathologies have various levels of comorbidities degrees. These observations clearly stress how complex are diagnostic and treatment modalities for the patients with a lung pathology. Therefore, advances in knowledge of pulmonary diseases thru interaction within inter- and multidisciplinary teams would have benefit in establishing most adequate and timely diagnostics, treatment and follow-up that will impact prognosis and quality of life in this group of patients. Furthermore, our findings will bring to the additional knowledge in optimal decision-making strategies for identification and referral of these patients to the defined professional specialist within a multiprofessional team for the purpose of treatment of both psychic and somatic comorbidities. This should have an influence on more effective treatment outcomes, thus reducing hospitalizations and medical costs.

## Figures and Tables

**Table 1:** Frequencies of specific characteristics of the patients.

Characteristics	Group 1 *N* = 120	Group 2 *N* = 74	Group 3 *N* = 38	Group 4 *N* = 40
Age (years) (MV ± SD)	66.22 ± 3.14	60.78 ± 2.99	54.24 ± 4.14	58.68 ± 4.09
Female/Male *N* (%)	51/69 (42.5%/57.5%)	31/43 (41.9%/58.1%)	23/15 (60.5%/39.5%)	16/24 (40%/60%)
Smoking (*N*%)	110 (91.7%)	67 (90.5%)	14 (36.8%)	36 (90%)
Hypertension (*N*%)	94 (78.3%)	55 (74.3%)	24 (63.2%)	34 (85%)
Obesity (*N*%)	45 (37.5%)	7 (9.5%)	15 (39.5%)	3 (7.5%)

**Table 2:** Values of HAM-A questions in different pathologies and comparisons among them.

HAM-A Questions	Group-1 MV ± SD (95% CI)	Group-2 MV ± SD (95% CI)	Group-3 MV ± SD (95% CI)	Group-4 MV ± SD (95% CI)	*p*
Anxious mood	1.25 ± 1.18 (1.04–1.46)	0.97 ± 1.01 (0.74–1.21)	0.87 ± 0.93 (0.56–1.18)	1.13 ± 1.11 (0.77–1.48)	0.349 *
Tension	1.54 ± 1.13 (1.34–1.75)	1.30 ± 0.98 (1.07–1.52)	1.26 ± 1.03 (0.92–1.60)	1.35 ± 1.21 (0.96–1.74)	0.438 *
Fears	1.12 ± 1.15 (0.91–1.33)	1.11 ± 1.09 (0.85–1.36)	0.84 ± 1.05 (0.50–1.19)	0.88 ± 0.97 (0.57–1.18)	0.469 *
Insomnia	1.69 ± 1.24 (1.47–1.92)	1.24 ± 1.14 (0.98–1.51)	1.32 ± 1.23 (0.91–1.72)	1.45 ± 1.15 (1.08–1.82)	0.097 *
Intellectual	0.81 ± 1.06 (0.62–1.00)	0.35 ± 0.75 (0.18–0.52)	0.45 ± 0.80 (0.19–0.71)	0.50 ± 0.85 (0.23–0.77)	0.031 *
Depressed mood	1.28 ± 1.25 (1.06–1.51)	0.86 ± 1.01 (0.63–1.10)	1.00 ± 0.99 (0.68–1.32)	0.85 ± 1.00 (0.53–1.17)	0.112 *
Somatic (muscular)	1.03 ± 1.21 (0.81–1.25)	0.46 ± 0.71 (0.30–0.62)	0.71 ± 0.93 (0.41–1.02)	0.85 ± 1.00 (0.53–1.17)	0.031 *
Somatic (sensory)	0.66 ± 1.04 (0.47–0.85)	0.34 ± 0.65 (0.19–0.49)	0.42 ± 0.89 (0.13–0.71)	0.65 ± 0.89 (0.36–0.94)	0.252 *
Cardiovascular symptoms	1.48 ± 1.21 (1.26–1.69)	0.65 ± 0.90 (0.44–0.86)	0.74 ± 0.95 (0.42–1.05)	0.98 ± 1.00 (0.66–1.29)	<0.001 *
Respiratory symptoms	2.37 ± 1.29 (2.13–2.60)	1.11 ± 1.08 (0.86–1.36)	1.11 ± 1.33 (0.67–1.54)	1.68 ± 1.27 (1.27–2.08)	<0.001 *
Gastrointestinal symptoms	1.30 ± 1.24 (1.08–1.52)	0.53 ± 0.78 (0.35–0.71)	0.58 ± 0.98 (0.26–0.90)	0.80 ± 1.02 (0.47–1.13)	<0.001 *
Genitourinary symptoms	0.67 ± 1.11 (0.47–0.87)	0.32 ± 0.72 (0.16–0.49)	0.39 ± 0.86 (0.11–0.68)	0.48 ± 0.88 (0.19–0.76)	0.342 *
Autonomic symptoms	0.64 ± 1.03 (0.46–0.83)	0.26 ± 0.60 (0.12–0.40)	0.55 ± 0.86 (0.27–0.84)	0.45 ± 0.93 (0.15–0.75)	0.134 *
Behavior at interview	0.78 ± 1.06 (0.58–0.97)	0.62 ± 0.95 (0.40–0.84)	0.47 ± 1.01 (0.14–0.80)	0.58 ± 0.87 (0.30–0.85)	0.411 *
Psychic subscale	8.47 ± 5.95 (7.39–9.54)	6.46 ± 4.71 (5.37–7.55)	6.21 ± 5.13 (4.52–7.90)	6.73 ± 5.53 (4.96–8.49)	0.029 **
Somatic subscale	8.14 ± 5.62 (7.13–9.16)	3.66 ± 3.73 (2.80–4.53)	4.50 ± 5.18 (2.80–6.20)	5.88 ± 5.23 (4.20–7.55)	<0.001 *
Total score	16.60 ± 10.55 (14.69–18.51)	10.12 ± 7.06 (8.49–11.76)	10.71 ± 9.68 (7.53–13.89)	12.58 ± 9.98 (9.38–15.77)	<0.001 **

* Kruskal–Wallis test; ** One way ANOVA test.

**Table 3:** Comparisons between HAM-A questions, subscales and total score in different pathologies.

HAM-A Questions	Group-1/ Group-2 (*p* Value)	Group-1/ Group-3 (*p* Value)	Group-1/ Group-4 (*p* Value)	Group-2/ Group-3 (*p* Value)	Group-2/ Group-4 (*p* Value)	Group-3/ Group-4 (*p* Value)
Intellectual	0.003 *	0.047 *	0.078 *	0.295 *	0.198 *	0.405 *
Somatic (muscular)	0.002 *	0.113 *	0.288	0.149 *	0.038 *	0.284 *
Cardiovascular symptoms	<0.001 *	0.001 *	0.013 *	0.337 *	0.046 *	0.142 *
Respiratory symptoms	<0.001 *	<0.001 *	0.002 *	0.323 *	0.013 *	0.023 *
Gastrointestinal symptoms	<0.001 *	<0.001 *	0.017 *	0.409 *	0.136 *	0.152 *
Psychic subscale	0.222 **	0.137 **	0.343 **	0.995 **	0.994 **	0.961 **
Somatic subscale	<0.001 *	<0.001 *	0.015 *	0.904 *	0.024 *	0.114 *
Total score	0.002 **	0.007 **	0.122 **	0.988 **	0.533 **	0.735 **

* Mann-Whitney U test; ** Post Hoc Tukey test.

**Table 4:** Correlation between HAM-A questions and different pathologies.

HAM-A Questions	Group-1		Group-2		Group-3		Group-4	
	r *	*p*	r *	*p*	r *	*p*	r *	*p*
Anxious mood	0.12	0.050	−0.07	0.234	−0.09	0.156	0.01	0.891
Tension	0.11	0.072	−0.06	0.306	−0.05	0.378	−0.02	0.716
Fears	0.06	0.310	0.04	0.535	−0.07	0.230	−0.06	0.303
Insomnia	0.15	0.011	−0.12	0.047	−0.06	0.364	−0.01	0.859
Intellectual	0.21	<0.001	−0.15	0.011	−0.06	0.319	−0.04	0.520
Depressed mood	0.17	0.005	−0.11	0.073	−0.02	0.698	−0.08	0.191
Somatic (muscular)	0.19	0.001	−0.20	<0.001	−0.04	0.549	0.02	0.770
Somatic (sensory)	0.12	0.051	−0.13	0.028	−0.05	0.400	0.05	0.396
Cardiovascular symptoms	0.32	<0.001	−0.23	<0.001	−0.12	0.046	−0.04	0.549
Respiratory symptoms	0.41	<0.001	−0.29	<0.001	−0.19	0.002	−0.02	0.721
Gastrointestinal symptoms	0.31	<0.001	−0.21	<0.001	−0.12	0.045	−0.04	0.479
Genitourinary symptoms	0.15	0.014	−0.12	0.054	−0.05	0.435	−0.01	0.817
Autonomic symptoms	0.14	0.018	−0.16	0.007	0.03	0.679	−0.02	0.725
Behavior at interview	0.10	0.096	−0.02	0.685	−0.08	0.210	−0.04	0.552
Psychic subscale	0.18	0.003	−0.10	0.105	−0.08	0.171	−0.05	0.440
Somatic subscale	0.34	<0.001	−0.27	<0.001	−0.12	0.051	−0.02	0.794
Total score	0.29	<0.001	−0.21	<0.001	−0.11	0.070	−0.04	0.564

* Point-Biserial Correlation.

## Data Availability

All data are presented in this study. Original data are available on reasonable request.
